# Āsana for Back, Hips and Legs to Prevent Musculoskeletal Disorders among Dental Professionals: In-Office Yóga Protocol

**DOI:** 10.3390/jfmk9010006

**Published:** 2023-12-22

**Authors:** Maria Giovanna Gandolfi, Fausto Zamparini, Andrea Spinelli, Carlo Prati

**Affiliations:** 1 Program in Ergonomics, Posturology and Yoga Therapy for the Degree in Dentistry and for the Degree in Dental Hygiene, School of Medicine, University of Bologna, 40125 Bologna, Italy; 2 Program in Yoga Therapy for the Specialization Course in Sports Medicine, School of Medicine, University of Bologna, 40125 Bologna, Italy; 3 Dental School, Department of Biomedical and Neuromotor Sciences, University of Bologna, 40125 Bologna, Italyandrea.spinelli4@unibo.it (A.S.); carlo.prati@unibo.it (C.P.)

**Keywords:** Yoga, Yoga Therapy, asana, musculoskeletal disorders, dental professionals, dental ergonomics, posture, low-back pain, spinopelvic mobility, iliopsoas syndrome, ischiofemoral impingement, *piriformis* syndrome, *quadratus femoris* dysfunction

## Abstract

Dental professionals are exposed to significant unavoidable physical stress, and theoretical ergonomic recommendations for a sitting workplace are inapplicable in many dental activities. Work-related musculoskeletal disorders (WMSDs) represent a serious health problem among dental professionals (prevalence: 64–93%), showing involvement of 34–60% for the low back and 15–25% for the hips. Muscle stress; prolonged sitting; forward bending and twisting of the torso and head; unbalanced working postures with asymmetrical weight on the hips and uneven shoulders; and others are inevitable for dental professionals. Therefore, the approach for the prevention and treatment of WMSDs must be therapeutic and compensatory. This project was conceived to provide a Yoga protocol for dental professionals to prevent or treat WMSDs from a preventive medicine perspective, and it would represent a Yoga-based guideline for the self-cure and prevention of musculoskeletal problems. Methods: Specific Yoga positions (āsana, such as *Virāsana, Virabhadrāsana, Garudāsana, Utkatāsana, Trikonāsana, Anuvittāsana, Chakrāsana, Uttanāsana, Pashimottanāsana)* have been selected, elaborated on and adapted to be practiced in a dental office using a dental stool or the dental office walls or a dental unit chair. The protocol is specifically devised for dental professionals (dentists, dental hygienists and dental assistants) and targeted for the low back, hips and legs (including knees and ankles). The protocol includes *Visranta Karaka Sthiti* (supported positions) in sitting (*Upavistha Sthiti*) and standing (*Utthistha Sthiti*) positions, twisting/torsions (*Parivrtta*), flexions/forward bend positions (*Pashima*) and extensions/arching (*Purva*) for musculo-articular system decompression and mobilization. Results: Over 60 Yogāsana—specifically ideated for back detensioning and mobilization, lumbar lordosis restoration, trunk side elongation, hip release and leg stretches and decontraction—are shown and described. The paper provides a meticulous description for each position, including the detailed movement, recommendations and mistakes to avoid, and the breathing pattern (breath control) in all the breath-driven movements (āsana in *vinyāsa*). An exhaustive analysis of posture-related disorders affecting the lower body among dental professionals is reported, including low-back pain, hip pain and disorders, *piriformis* syndrome and *quadratus femoris* dysfunction (gluteal pain), *iliopsoas* syndrome, *multifidus* disorders, femoroacetabular and ischiofemoral impingement, spinopelvic mobility, lumbopelvic rhythm, impairment syndromes, lower crossed syndrome, leg pain, knee pain and ankle disorders. Conclusions: A detailed guideline of āsana for low-back decompression, hip joint destress, *piriformis* and gluteal muscle release, lumbar lordosis recovery and a spinopelvic mobility increase has been elaborated on. The designed Yogāsana protocol represents a powerful tool for dental professionals to provide relief to retracted stiff muscles and unbalanced musculoskeletal structures in the lower body.

## 1. Introduction

Work-related musculoskeletal disorders (WMSDs) are serious health problems among dental professionals, showing a prevalence ranging from 64% to 93% [1,2,3,4]. The body areas most affected by WMSDs among dental professionals are the low back (34–60%) and hips (15–25%) in addition to the neck and shoulders [3,5,6,7,8,9]. Many studies from top-ranked medical journals report that the low back and hip are areas highly affected by musculoskeletal disorders among dental professionals [3,4,5,10,11,12]. 

Low-back and hip pain include spine sagittal imbalance and loss of lumbar lordosis, lumbopelvic rhythm alteration, lower crossed syndrome, compressive syndromes and neuropathies including lumbar disk herniation, *piriformis* syndrome, hip disorders, gluteal pain, lateral hip pain and arthrosis [3]. 

We need to highlight that prolonged and/or inadequate sitting postures induce a reduction in spinopelvic mobility, muscle stiffening and straining often associated with weakening and are strongly related to spinopelvic syndromes caused by alterations of lumbopelvic biomechanics and retraction/stiffness of muscles and soft tissues in lumbar and hip areas. In addition, non-physiological sitting behavior (as loss of lumbar lordosis, kyphotic sitting posture, forward trunk inclination, anterior pelvic tilt) and postural behavior in kinetics during movement causes the increase in strains and loads on the back with a consequent enhanced risk for postero-lateral disk pathologies (bulging, protrusion, herniation) and compression of the abdominal (and thoracic) organs. 

Indeed, extended literature reports that a protracted sitting posture is correlated with many health problems, from cardiovascular pathologies [13] and respiratory disorders [14] to musculoskeletal (compressive or impairment) syndromes including low-back pain [15,16,17], spine mobility [18], hip extension [19], pelvic tilt [20] and lumbar lordosis [14], *iliopsoas* [21] and *piriformis* [22] syndromes and hamstring tightness [23]. We will address these topics in detail in the discussion.

The risk factors for sitting behavior elaborated on following Yoga Therapy principles are summarized in Table 1. 

Ergonomics aim to reduce or eliminate stress, injuries and disorders by controlling ergonomic hazards and worker exposure to work-related musculoskeletal disorder risk factors. However, most of the risk factors cannot be eliminated nor reduced due to the inevitable demand for muscle overuse, repetitive and prolonged movements and an unbalanced posture required by dental practice.

In addition, ergonomic recommendations for a sitting position are theoretical rules inapplicable in many dental activities. Specifically, we refer to the muscular stress and muscle overuse (repetitive movements, forced and forceful maneuvers, mechanical compression, prolonged and frequent vibrations), forward bending, head and/or torso twist, asymmetrical weight on hips and uneven shoulders and more. Stressful activities and repeated tasks cause muscle weakness/soreness/spasms/cramps, muscle injury (and swelling with frequent nerve pinching or compression, with possible onset of paresthesia and tingling), muscle pain, loss of muscular and articular mobility and formation of trigger points [3].

As addressed and discussed in depth in a recent paper [9], a wrong posture is often unperceived and then uncontrollable or is unavoidable. Then, the authors on many occasions highlighted the crucial need to train dental professionals and mainly dental students to cultivate the (unconscious) perception of body postures and movements, reducing the unperceived or involuntary activities. To this purpose, Yoga as *concentrative practice* creates a continuous body–mind connection developing an “*embodied mind*”.

Yoga is a system of physical, mental and spiritual techniques/practices providing holistic well-being [9]. 

Yoga as therapeutic science received increasing attention over the past decades and has been an object of study with a high number of scientific publications in different medical disciplines, demonstrating great therapeutic effects and benefits for both physical and mental/cognitive health. A large number of clinical studies on Yoga as a medical approach for different pathologies science have been published in highly rated/top-scored journals in various medical fields, specifically in cardiology for cardiovascular diseases [24,25,26], coronary heart disease [27], hypertension [28], atrial fibrillation and arrhythmia [29] and vasovagal syncope [30]; in internal medicine for metabolic syndrome [31,32], irritable bowel [33], inflammatory markers [34,35] and immune system [36]; in neurology/psychology for stress, anxiety and burnout [37,38]; in pneumology [39,40]; in orthopedics/physiatry for chronic pain and pain-associated disorders [41,42,43,44,45,46,47,48,49,50,51,52] and disc herniation [45,46,47]; in occupational and preventive medicine and public health [37,48]. 

This paper aims to ideate and describe a Yoga protocol for dental professionals consisting of āsana that can be performed in a dental office using a dental stool, a dental unit chair or the dental office walls. The proposed postures and movements have been conceived to mobilize and decompress the musculo-articular system in the low back, hip and leg/ankle, being areas greatly affected by musculoskeletal disorders. 

## 2. Materials and Methods

A Yogāsana protocol specifically ideated for dental professionals and focused on the mobilization, muscle release and articular decompression/loosening up of the low back, hips and legs is described and shown. 

The project was born from over 10 years of experience of teaching Posturology to university students of dental disciplines. The study is part of a Yoga project approved on 25th March 2022 by the regional remit ethical committee (approval protocol number: 847/2021/OSS/AUSLBO).

### 2.1. Targeted Body Areas

The protocol elaborated on selected āsana and ideated Yoga postures and movements for dental professionals (dentists, dental hygienists and dental assistants) finalized to be practiced using the structures present in the dental office (dental chair, clinic/office walls and furniture). 

The targeted areas of the devised āsana concern the lower part of the body, specifically the low back and intervertebral articulations, hip joint, sacroiliac joint and legs including the knee joint and ankle joint, and the release of the involved muscles such as the *iliopsoas*, *piriformis*, *quadratus femoris*, *multifidus* and *quadratus lumborum*.

For each āsana, the origin (O) and insertion (I) of the involved muscles are reported to understand the rationale of the movement rules of our Yoga protocol. The authors theory is that the visualization of muscle attachments facilitates the perception of the movement that must be performed to obtain health benefits. The protocol is based on joint biomechanics science and on the theory of the “bone ties” (joint compressions caused by bone-to-bone tractions through musculo-tendinous-ligamentous system) elaborated on and elucidated in our previous article [9]. The ideated Yogāsana release the bone ties and decompress the joints. 

### 2.2. Āsana (Yoga Postures)

The āsana have been selected, elaborated on and adapted to be practiced/performed in a dental office using a dental stool or the dental office walls or a dental unit chair. The protocol is specifically devised for dental professionals, namely dentists, dental hygienists and dental assistants to mobilize and decompress the musculoskeletal structures during their daily routine.

Yogāsana have been ideated and showed in the images by one of the authors (M.G.G.), being a certified experienced Yoga teacher with over 20 years of daily self-practice, and university professor of Posturology and Ergonomics and professor of Yoga Therapy since 2012.

Āsana names were mainly derived and elaborated on from *Hathayoga Pradipīkā* (XV century), *Krishnamacharya Yoga* (Tirumalai Krishnamacharya 1934, Yoga-Makaranda), *Iyengar Yoga* style (Bellur Krishnamachar Sundararaja Iyengar 1966, The Light on Yoga) and others (Swami Satyananda Saraswati 1969, Satyananda-Yoga; Rishikesh Yoga derived by Sivananda Yoga and elaborated on in Swami Satyananda sequences). 

Āsana practice is rooted and elaborated on from *Krishnamacharya Yoga*, *Iyengar Yoga* and *Parināma Yoga* techniques.

The illustrated positions, grouped per body area, are listed below. The protocol includes *Visranta Karaka Sthiti* (supported positions) in sitting, *Upavistha Sthiti* (seated positions) and standing positions (*Utthistha Sthiti*), with twisting/torsions (*Parivrtta*), flexions/forward bend positions (*Pashima*) and extensions/arching (*Purva*) for musculo-articular decompression and mobilization. The protocol is contrived with a rationale of movements in all directions of space, with gradually increasing intensity.

All Yogāsana are “holistic” postures and the movement involves the musculo-articular system in more than one area, such as the back and shoulders or back and legs or hips and legs.

 

***Āsana with backward bending for back extension, spine mobilization and lordosis recovery:***

- *Upavistha Anuvittāsana* (Seated Backbend Pose)
- *Bhujangāsana* (Cobra Pose)
- *Urdhva Mukha Svanāsana* (Upward Facing Dog)
- *Anuvittāsana* (Standing Backbend Pose)
- *Chakrāsana* or *Urdhva Dhanurāsana* (Full-Wheel Pose or Upward Bow Pose)
- *Dwi Pada Viparita Dandāsana* (Two-Legged Inverted Staff Pose)

 

***Āsana for trunk side and twists (trunk side outstretch, trunk torsion, leg stretching, hip distraction and glide):***

- *Parsva Urdhva Hastāsana* (Standing Half Moon Pose or Upward Salute Side Bend Pose)
- *Bananāsana* (Crescent Moon Pose)
- *Trikonāsana* (Triangle Pose)
- *Parivrtta Trikonāsana* (Twisted Triangle Pose)
- *Jathara Parivartanāsana* (Belly Twist A or Seated Spinal Twist A) or *Meru Wakrāsana* (Seated Twist)
- *Parivrtta Utkatāsana* (Twisting Chair Pose)
- *Parivrtta Utkata Konāsana* (Revolved Goddess Pose)

 

***Āsana with forward bending for back release:***

- *Uttanāsana* (Forward Fold Pose)
- *Pashimottanāsana* (Seated Forward Bend Pose)
- *Parivrtta Pashimottanāsana* (Revolved Seated Forward Bend Pose)
- *Adho Mukha Svanāsana* (Downward Facing Dog)
- *Upavistha Dwikonāsana* (Seated Double-Angle Pose or Yoga Seal Pose)

 

***Āsana for hip flexors and hip release:***

- *Eka Pada Supta Virāsana* (One-Legged Reclined Hero Pose)
- *Parivrtta Virabhadrāsana I* (Revolved Warrior I) or *Parivrtta Ashva Sanchalāsana* (Revolved High Lunge Pose)
- *Ashva Sanchalāsana* (High Lunge Pose)
- *Virabhadrāsana I* (Warrior I)
- *Valakhilyāsana* (Heavenly Spirits Pose)
- *Virabhadrāsana II* (Warrior II)
- *Viparita Virabhadrāsana* (Reverse Warrior) or *Parighāsana* (Gate Pose)
- *Garudāsana* (Eagle Pose)
- *Eka Pada Agnistambhāsana* (One-Legged Firelog or Square or Pigeon Pose)
- *Sucirandhrāsana* (Eye of the Needle Pose or Figure-4 Stretch)
- *Eka Pada Parivrtta Utkatāsana* (One-Legged Revolved/Twisted Chair Pose)

 

### 2.3. Execution Instructions and Recommendations

Āsana are positions you assume in which you need to be present, with the body and mind, as Yoga connects/unites and integrates the body and mind, as Yoga subjugates/yokes the mind and stills mind fluctuations.

The authors recommend—as a foundation of postures in Yoga Therapy—that movements be aware, slow and progressive, always performed by setting the intention to activate the body thrust in three spatial directions (three-dimensional push) with the intention of steady active pushing in three divergent directions (three-dimensional away urges, push intentionality in three directions).

We want to provide the concept that active pushing is basically different from active stretching.

Fundamental recommendations developed by the authors are described in detail below.

***Overall recommendations*** for performing the proposed āsana are to start with a balanced sitting position (same weight on ischial tuberosities, i.e., on both gluteus maximus) with the spinal curves maintained. Start moving slowly into the āsana after a deep breath (with full lungs) and continue to deepen the movement during the exhalation. Begin the movement from the low back (without sliding the sit bones on the seat) and proceed upward along the spine through the thoracic and cervical spine and finally the head. 

Remember that the condition with full lungs favors a correct/safe sitting posture and allows to avoid movement in a state of compression. Search for abdominal breathing and avoid apnea. Relax your eyes, cheeks and mandible (keep your mouth closed and breathe through your nose).

A Yoga protocol entails that the practice of the āsana is in a state of concentration and in *vinyāsa* (movements flowing with breath). Āsana must be always practiced in *vinyāsa*, the Yoga method which consists of the movement synchronous with the breath, i.e., the movement flows with breath and increases in intensity with breath. 

Enter into each āsana slowly and gently, possibly with closed or half-closed eyes and the intention to stretch away, to extend, to expand outward by a three-dimensional deep extension. Create the expansion by pushing (not pulling) your limbs away. For each inhalation, search for an increase in your inner space; for each exhalation, search for an extension in the stretch. 

Yoga postures can be performed in static or in slow dynamics. 

Keep a serendipity mindset, i.e., remain open and receptive to body messages, and welcome every unexpected sensation and feeling without resistance or opposition or judgement.

If the tension is painful or too strong, keep your focus on even slow breathing as if you had to melt into the position and if necessary, reduce the stretch intensity. A main teaching from intense āsana is to train the mind to be calm and still in a difficult/uneasy or painful condition and to be comfortable in discomfort. 

The authors suggest short breaks (at mid-morning, at lunch time, at mid-afternoon and at the end of the working day) to restore a strained, stiff and tired low back, hip and legs.

The authors recommend to reserve a mental space to practice Yoga postures, and to remain present and attentive in the body and breath.

***Recommendations for twisting***—The authors advise that the spine must be perpendicular to the floor during the entire twisting movements. Only at the end of the torsion the bending can start.

Start moving slowly into the rotation after a deep breath (with full lungs) and continue to increase the amount of torsion during the exhalation. Begin the twist from the low back (without sliding the sit bones on the seat) and proceed upward along the spine through the thoracic and cervical spine and lastly rotate the head. 

***Recommendations for forward bending***—Perceive a balanced position (in sitting position, same weight on ischial tuberosities, i.e., on both gluteus maximus) with the spinal curves maintained. Take a deep inhalation, then start moving slowly into the āsana and continue to deepen the movement during the exhalation. Begin the movement from the hips and proceed upward along the spine through the low back and the thoracic and cervical spine up to the head. 

***Recommendations for back bending (extensions)***—From a balanced position (in sitting position, same weight on ischial tuberosities, i.e., on both gluteus maximus), take a deep inhalation. Slightly engage deep core muscles, mainly the *transversus abdominis* and pelvic floor muscles (feel the activation of the *puboanalis* muscles and anal and urethral sphincters), then start moving slowly into the extension and continue to deepen the back bend during the exhalation. If in a standing position, begin the movement with hip counternutation (the sacrum is rotated backwards relative to the iliac bones) and proceed upward along the spine through the low back, the thoracic and cervical spine and, only at the end, the head.

## 3. Results

**Figure 1:**
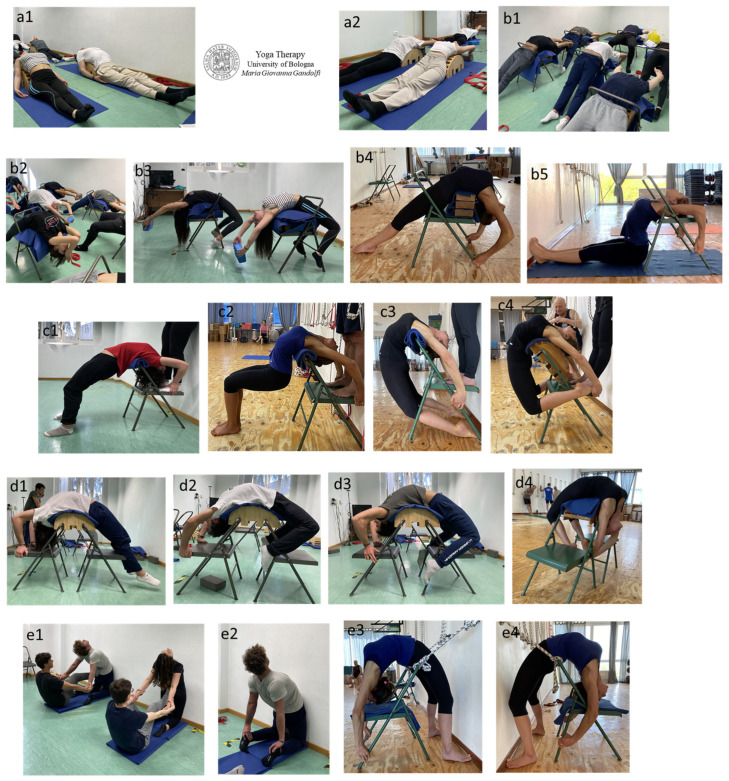
The figure shows Yoga Therapy āsana practiced by students of the Degree in Dentistry and Degree in Dental Hygiene and by Medical Doctors specializing in Sports Medicine at the University of Bologna. Specific āsana for low-back mobilization (**a1**,**a2**,**b1**–**b3**,**c1**,**d1**–**d3**) and hip flexor release (**e1**,**e2**) are displayed. Advanced āsana (**b4**,**b5**,**c2**–**c4**,**d4**,**e3**,**e4**) practiced by their university professor and experienced Yoga practitioner are illustrated.

Figure 1 shows students of the Yoga Therapy university courses practicing specific āsana for low-back mobilization (Figure 1(a1,a2,b1–b3,c1,d1–d3)) and hip flexor release (Figure 1(e1,e2)). Advanced āsana are illustrated in Figure 1(b4,b5,c2–c4,d4,e3,e4). 

We would like to highlight that our university Yoga classes at the Dental School contemplate a large number of āsana and movements for the spine and hips in supine (*supta*) and prone (*advarjita*) and standing (*utthistha*) positions, by also using props, supports and ropes (*rajju* āsana) and following the *Hatha Yoga* technique and its evolutions following a therapeutic approach to the body (*Iyengar Yoga* and *Parināma Yoga*).

**Figure 2:**
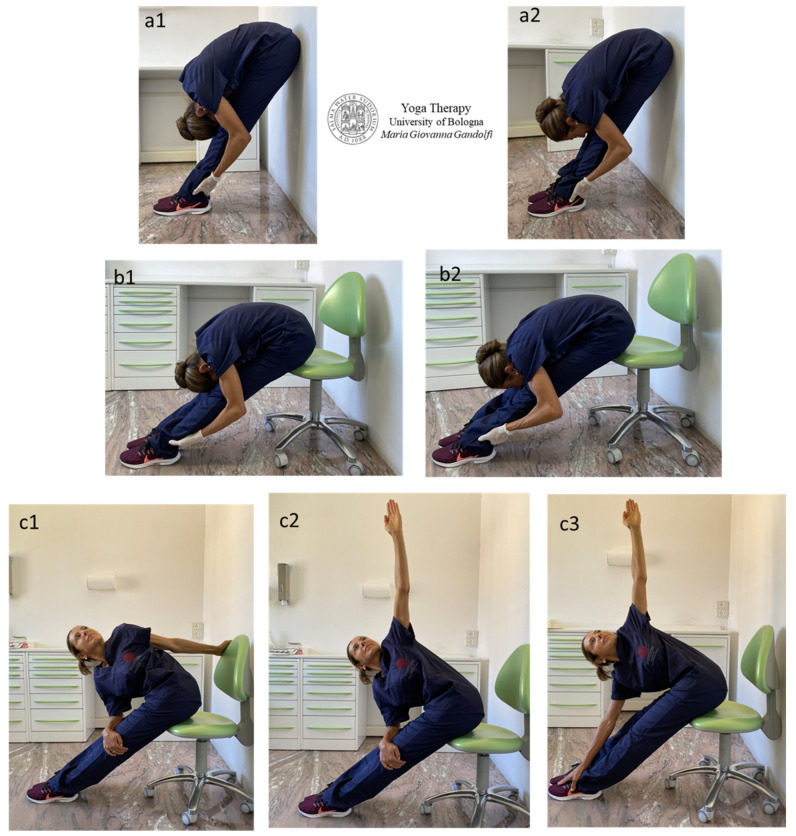

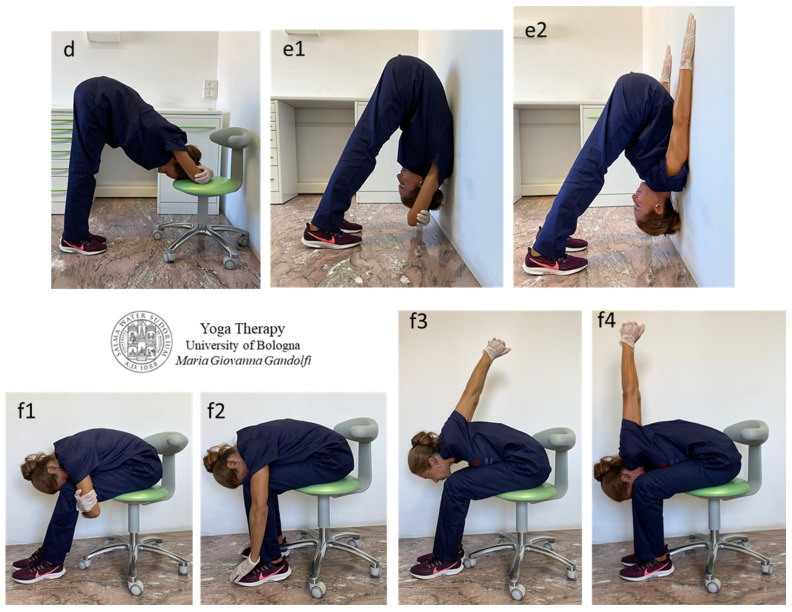
The images display *Uttanāsana* (Forward Fold Pose) (**a1**,**a2**), *Pashimottanāsana* (Seated Forward Bend Pose) (**b1**,**b2**), *Parivrtta Pashimottanāsana* (Revolved Seated Forward Bend Pose) (**c1**–**c3**), *Adho Mukha Svanāsana* (Downward Facing Dog) (**d**), *Uttanāsana* (Forward Bend) (**e1**,**e2**) with *Baddha Bhuja* (bound arms) and wall support (**e1**) and *Upavistha Dwikonāsana* (Seated Double-Angle Pose or Yoga Seal Pose) (**f1**–**f4**) with *Baddha Hasta* (**f3**,**f4**) positions.

Figure 2 shows movements to release strained and sore muscles of the back part of the body. The described āsana are *Uttanāsana* (Forward Fold Pose) (Figure 2(a1,a2)), *Pashimottanāsana* (Seated Forward Bend Pose) (Figure 2(b1,b2)), *Parivrtta Pashimottanāsana* (Revolved Seated Forward Bend Pose) (Figure 2(c1,c3)), *Adho Mukha Svanāsana* (Downward Facing Dog) (Figure 2d), *Uttanāsana* (Forward Bend) variation with wall support (Figure 2(e1,e2)) and with *Baddha Bhuja* (bound arms) (Figure 2(e1)), *Upavistha Dwikonāsana* (Seated Double-Angle Pose or Yoga Seal Pose) (Figure 2(f1–f4)).

The displayed bending movements stretch out the back muscles of the trunk including the *erector spinae* group (*iliocostalis*, *longissimus and transversospinalis* enclosing *multifidus* and *rotatores*) and *quadratus lumborum*, outspread the posterior leg muscles such as the hamstrings and calf muscles (*popliteus*, *gastrocnemius* and *soleus*) and mobilize the sacroiliac joint and de-tension its stabilizing muscles such as *piriformis* and *gluteus* muscles.

The authors want to attract attention to the different movement and back shape in Figure 2(a1) vs. 2(a2) and Figure 2(b1) vs. 2(b2). In Figure 2(a1,b1) (forehead on knees), the back is rounded and the stretch involves the *erectus spinae*, mainly the *multifidus* and *quadratus lumborum*. This execution of *Uttanāsana* and *Pashimottanāsana* is practiced in *Krishnamacharya Yoga* (Krishnamacharya in Yoga-Makaranda, 1934) and other styles (such as Swami Satyananda Saraswati in Satyananda-Yoga, 1969; Rishikesh Yoga derived by Sivananda Yoga and elaborated on in Swami Satyananda sequences; etc.). Differently, in Figure 2(a2,b2), the chin is over the knees and the back is straight with stretch and traction intention. The elongation mainly involves the posterior muscles of the leg. This practice of *Uttanāsana* and *Pashimottanāsana* follows the *Parināma Yoga* technique and *Iyengar Yoga* style (Iyengar in The Light on Yoga, 1966).

**Figure 3:**
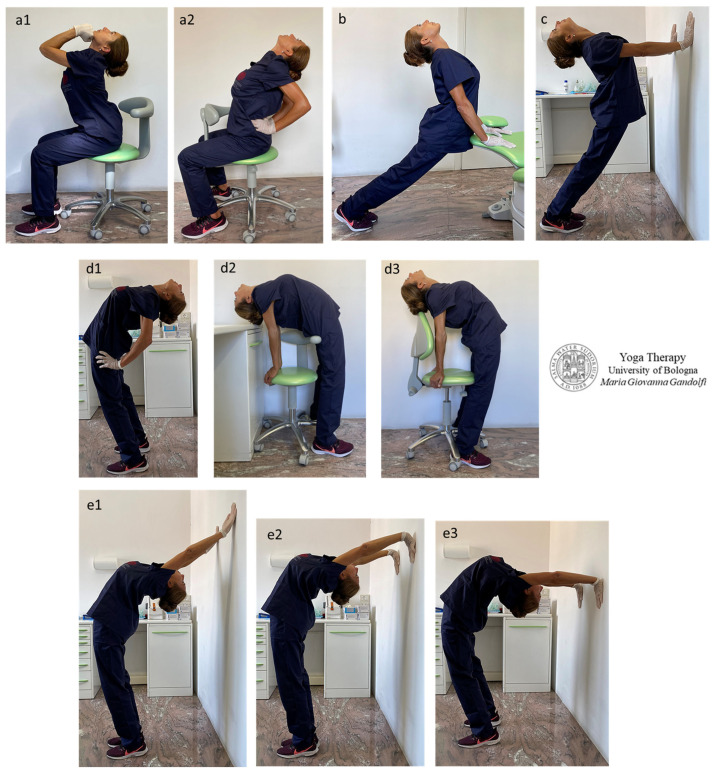

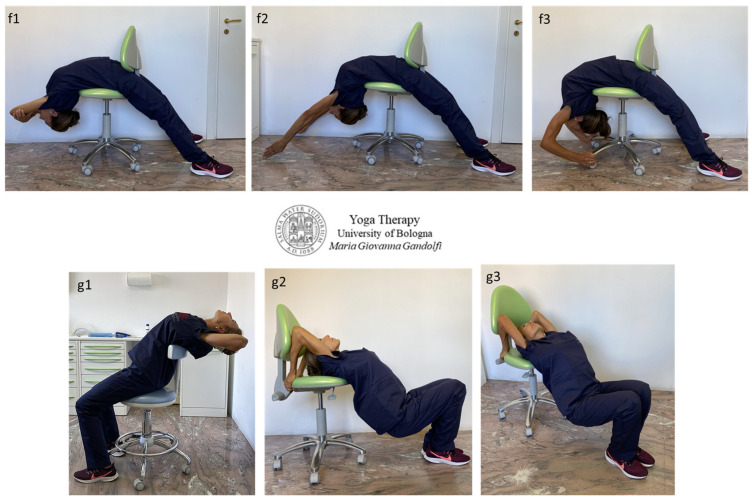
The images display *Upavistha Anuvittāsana* (Seated Backbend Pose) with *Mushti Mudra* (hands in fists) pushing the chin backwards to increase neck extension (**a1**) or hand palms favoring low-back extension (**a2**), *Bhujangāsana* (Cobra Pose) (**b**), *Urdhva Mukha Svanāsana* (Upward Facing Dog) standing (*Utthistha*) variation (**c**), *Anuvittāsana* (Standing Backbend Pose) and variations (**d1**–**d3**), *Chakrāsana* or *Urdhva Dhanurāsana* (Full-Wheel Pose or Upward Bow Pose) (**e1**–**e3**), *Dwi Pada Viparita Dandāsana* (Two-Legged Inverted Staff Pose) (**f1**–**f3**) and its preparations (**g1**–**g3**).

Figure 3 illustrates compensatory Yogāsana elaborated on to mobilize and decompress the lumbar spine and restore spinal lordosis in the lumbar region and in the neck.

Āsana in Figure 3 display *Upavistha Anuvittāsana* (Seated Backbend Pose) with hands in *Mushti Mudra* (Fist Gesture) who put pressure against the chin to favor neck extension (Figure 3(a1)) or hand palms are pushing the sacrum to favor low-back extension (Figure 3(a2)), *Bhujangāsana* (Cobra Pose) (Figure 3b), *Urdhva Mukha Svanāsana* (Upward Facing Dog) standing (*Utthistha*) variation (Figure 3c), *Anuvittāsana* (Standing Backbend Pose) and variations (Figure 3(d1–d3)), *Chakrāsana or Urdhva Dhanurāsana* (Full-Wheel Pose or Upward Bow Pose) (Figure 3(e1–e3)), *Dwi Pada Viparita Dandāsana* (Two-Legged Inverted Staff Pose) (Figure 3(f1–f3)) and its preparations (Figure 3(g1–g3)).

The Authors highlight that āsana with actively kept straight legs and involving counternutation of the sacroiliac joint provide a deep elongation and release of hip flexors, mainly the *iliopsoas*, *sartorius* and *pectineus muscles.*

Āsana with deep chest extension (mainly Figure 2(d2,d3,e1–e3,f1–f3)) strongly stretch out *intercostal* and *subcostal muscles*, impose a diaphragmatic breathing and provide the decompression of abdominal organs.

**Figure 4:**
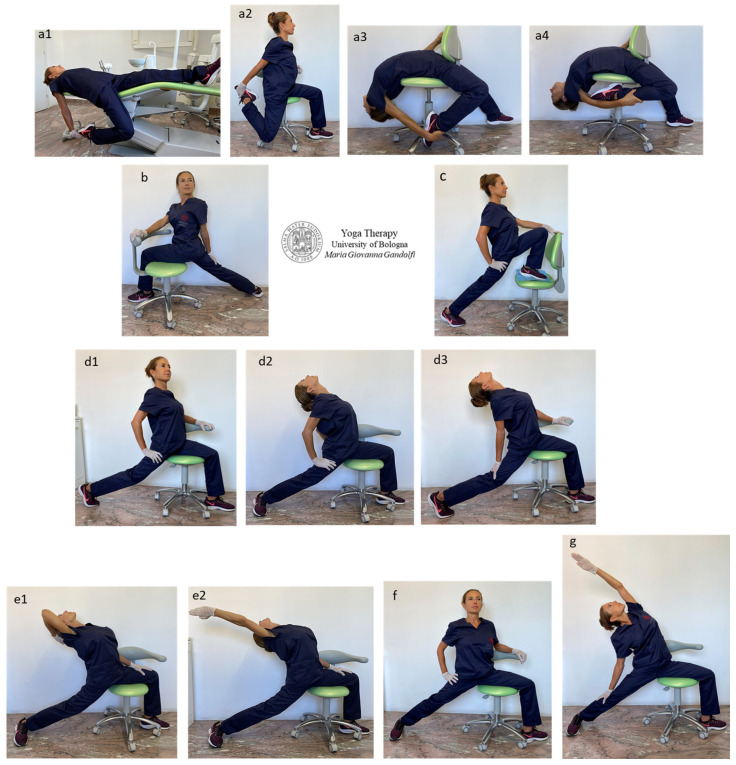
The figure shows devised Yogāsana with chair variations, namely *Eka Pada Supta Virāsana* (One-Legged Reclined Hero Pose) (**a1**–**a4**) with *Supta* variation (**a1**), *Parivrtta Virabhadrāsana I* (Revolved Warrior I) or *Parivrtta Ashva Sanchalāsana* (Revolved High Lunge Pose) (**b**), *Ashva Sanchalāsana* (High Lunge Pose) (**c**), *Virabhadrāsana I* (Warrior I) (**d1**–**d3**), *Valakhilyāsana* (Heavenly Spirits Pose) standing (*Utthistha*) variation (**e1**,**e2**), *Virabhadrāsana II* (Warrior II) (**f**), *Viparita Virabhadrāsana* (Reverse Warrior) or *Parighāsana* (Gate Pose) variation on chair (**g**).

Figure 4 shows āsana variations on a chair devised by the authors mainly for hip extension, but also involving the anterior leg muscles elongation and back extension.

The illustrated Yogāsana are *Eka Pada Supta Virāsana* (One-Legged Reclined Hero Pose) (Figure 4(a1)), *Eka Pada Virāsana* (One-Legged Seated Hero Pose) (Figure 4(a2–a4)), *Parivrtta Virabhadrāsana I* (Revolved Warrior I) or *Parivrtta Ashva Sanchalāsana* (Revolved High Lunge Pose) (Figure 4b), *Ashva Sanchalāsana* (High Lunge Pose) (Figure 4c), *Virabhadrāsana I* (Warrior I) (Figure 4(d1–d3)), *Valakhilyāsana* (Heavenly Spirits Pose) standing (*Utthistha*) variation (Figure 4(e1,e2)), *Virabhadrāsana II* (Warrior II) (Figure 4f), *Viparita Virabhadrāsana* (Reverse Warrior) or *Parighāsana* (Gate Pose) variation on a chair (Figure 4g). 

The *quadriceps femoris* muscles are involved in the shown ideated āsana on the chair to stretch the anterior muscles of the thigh, namely the *rectus femoris* (O: anterior inferior iliac spine; I: tibial tuberosity via patellar ligament), *vastus lateralis* (O: intertrochanteric line, greater trochanter, gluteal tuberosity; I: tibial tuberosity via patellar ligament, patella, lateral condyle of tibia), *vastus medialis* (O: intertrochanteric line, pectineal line of femur; I: tibial tuberosity via patellar ligament, patella, medial condyle of tibia), *vastus intermedius* (O: anterior surface of the upper part of femoral diaphysis; I: tibial tuberosity via patellar ligament, patella), *tensor vastus intermedius* (O: anteroinferior aspect of the greater trochanter; I: patellar base).

Specifically, the *rectus femoris* and the other *quadriceps femoris* muscles, namely the *vastus lateralis*, *medialis* and *intermedius* and *tensor vastus intermedius*, are stretched by knee flexion and mainly when knee flexion is associated with leg extension (Figure 4(a1–a4)).

The muscles mainly involved in the shown Yogāsana for hip extension are the *psoas major* (O: (superficial part) sides of T12–L4 including the intervertebral discs, (deep part) transverse processes of L1–L5; I: lesser trochanter of the femur), *psoas minor* (O: vertebral bodies of T12 and L1; I: below the inferior iliac spine on the iliopectineal eminence (junction of ilium–pubis bones) and *iliacus* (O: superior two-thirds of the iliac fossa and lateral aspect of the sacrum and anterior sacroiliac and iliolumbar ligaments; I: lesser trochanter of the femur), *pectineus* (O: superior pubic ramus (pectineal line); I: below the lesser trochanter of femur posterior surface) and both the *sartorius* (O: anterior superior iliac spine; I: medial side of the proximal tibia) and *tensor fascia latae* (O: anterior superior iliac spine; I: iliotibial band).

In particular, the *psoas* and *iliacus (iliopsoas*) muscles are outstretched when one straight leg is positioned posteriorly, with synchronous pelvis counternutation, core activation and the spine (initially) perpendicular to the ground. Different antero-medial hip muscles are involved when the back foot is resting on the metatarsals with a raised heel (Figure 4(d1,d3)) or the foot is rotated at 45 degrees with the heel resting on the ground (Figure 4(d2,e1,e2)). The *pectineus*, *abductors* and *gracilis* muscles are particularly involved when the posterior foot is rotated at 45 degrees and the heel is grounded (Figure 4(d2,e2)), and the *iliopsoas* and *sartorius* together with the *rectus femoris* are intensely stretched with a straight posterior leg and foot firmly recumbent on metatarsal bones.

 

**Figure 5:**
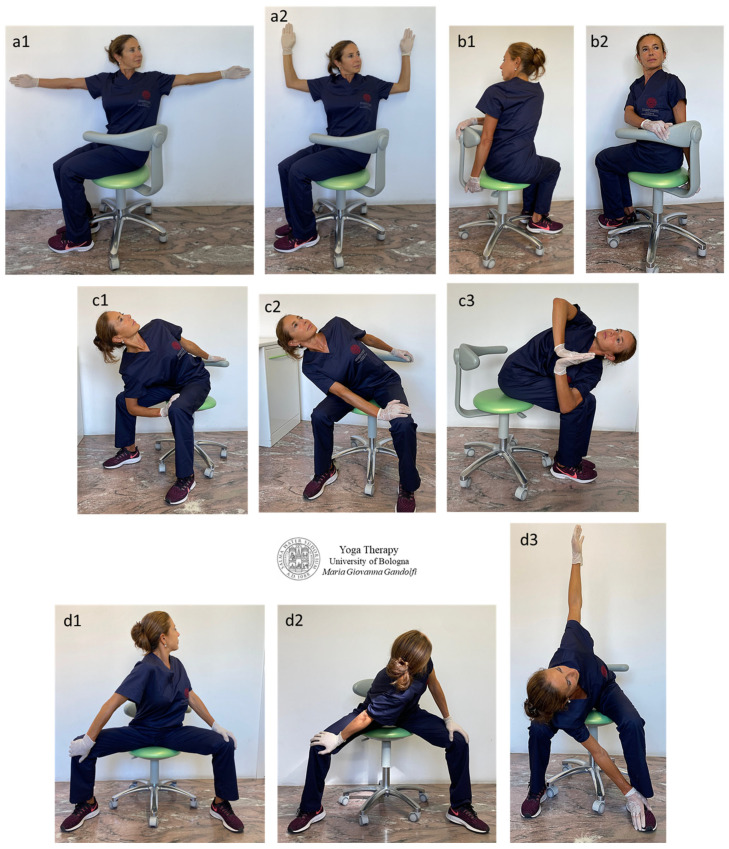
The figure shows the proposed āsana to release and decontract paraspinal muscles by trunk twisting (**a1**,**a2**,**b1**,**b2**,**d1**) and also twist associated with bending (**c1**–**c3**,**d2**,**d3**). *Jathara Parivartanāsana* (Belly Twist A or Seated Spinal Twist A) seated variation or *Meru Wakrāsana* (Seated Twist) (**a1**,**a2**,**b1**,**b2**), *Parivrtta Utkatāsana* (Twisting Chair Pose) (**c1**–**c3**) and *Parivrtta Utkata Konāsana* (Revolved Goddess Pose) seated variations (**d1**–**d3**).

Figure 5 shows āsana involving trunk twisting selected to stretch strained and stiff back muscles such as the erector spinae and quadratum lumborum. These include *Jathara Parivartanāsana* (Belly Twist A or Seated Spinal Twist A) seated variation or *Meru Wakrāsana* (Seated Twist) (Figure 5a–d), *Parivrtta Utkatāsana* (Twisting Chair Pose) (Figure 5(c1–c3)) and *Parivrtta Utkata Konāsana* (Revolved Goddess Pose) seated variations (Figure 5(d2,d3)).

The displayed twisting movements stretch out the paraspinal (or paravertebral) muscles—including the erector spinae group (*iliocostalis*, *longissimus and transversospinalis* enclosing *multifidus* and *rotatores*) and *quadratus lumborum*—muscles surrounding and attaching to the rachis and responsible for spine movement and stabilization.

 

 

The spine twist mainly releases the *iliocostalis lumborum* (O: medial and lateral sacral crests; medial iliac crest, thoracolumbar fascia, spinous processes of vertebrae from T11–L5; I: ribs 7–12), *multifidus* (O: posterior sacrum, posterior superior iliac spine, posterior sacroiliac ligament, mammillary processes of lumbar vertebrae, transverse processes of thoracic vertebrae and superior articular processes of C4–C7; I: spinous processes of vertebrae (except C1) 2–5 bones above origin), *rotatores* (O: transverse processes; I: bases of spinous processes of adjacent vertebrae in *r. brevis* or of two vertebrae above in *r. longus*), *intertransversarii* (lumborum, thoracis and cervicis) (O ↔ I: between the transverse processes of consecutive vertebrae) and *interspinales* (lumborum, thoracis and cervicis) (O ↔ I: between the spinous processes of adjacent vertebrae) muscles.

When the bending is added to torsion (Figure 5(c1,c3,d2,d3)), there is an increase in *iliocostalis lumborum*, *multifidus* and *intertransversarii* and *multifidus* outstretching and an additional stretch of the *quadratus lumborum* (O: posterior iliac crest; I: transverse processes of L1–L4 and rib 12) and *longissimus toracis*, and *rotatores lumborum* and *thoracis*.

The bending movement mainly acts on the muscles attached to the sacrum and/or iliac crest and to transverse and spinous processes of the vertebrae (and to the ribs). These muscles are the *multifidus*, *quadratus lumborum*, *iliocostalis lumborum*, *longissimus thoracis* and *spinalis thoracis*. The side bending is useful to soften the stiff *intertransversarii* and *rotator* muscles. Recommendations for performing these āsana are to start with a balanced sitting position (same weight on ischial tuberosities, i.e., on both *gluteus maximus*) with the spinal curves maintained. The spine must be perpendicular to the floor (Figure 5(b2,d1)) during the entire twisting movement. Only at the end of the torsion the bending can start (Figure 5(c1–c3,d2,d3)).

Start moving slowly into the rotation after a deep breath (with full lungs) and continue to increase the amount of torsion during the exhalation. Begin the twist from the low back (without sliding the sit bones on the seat) and proceed upward along the spine through the thoracic and cervical spine and lastly rotate the head. 

The condition with full lungs favors a correct/safe sitting posture and allows to avoid movement in a state of compression.

 

 

**Figure 6:**
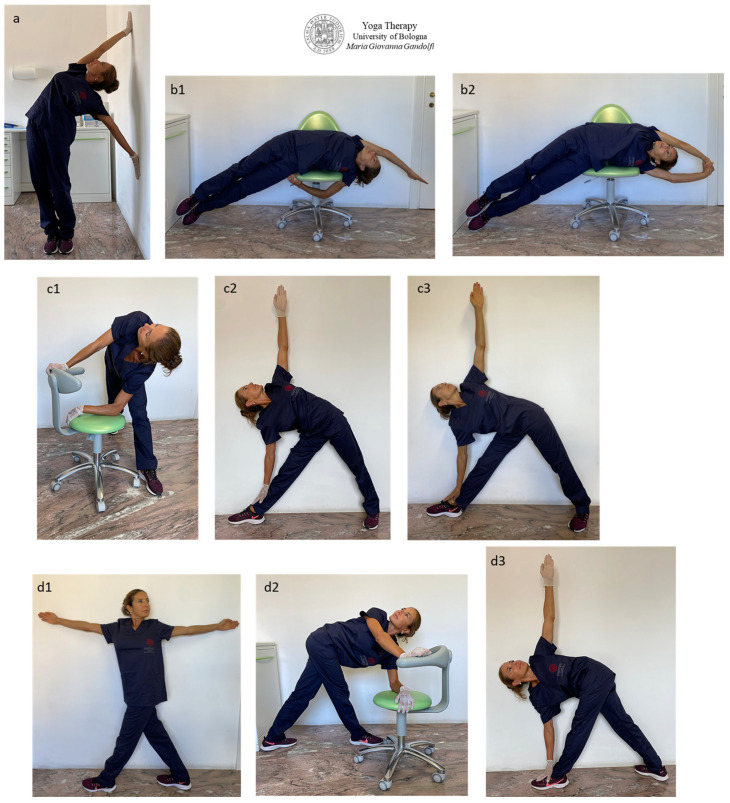
The figure illustrates *Parsva Urdhva Hastāsana* (Standing Half Moon Pose or Upward Salute Side Bend Pose) (**a**) and *Bananāsana* (Crescent Moon Pose) variations lying on the side (**b1**,**b2**), *Trikonāsana* (Triangle Pose) (**c1**–**c3**) with extended (*Utthita*) variation (**c3**), *Parivrtta Trikonāsana* (Twisted Triangle Pose) (**d1**–**d3**) for trunk side outstretch, trunk torsion and leg stretching. Hip glide (and therefore hip release) is intense in the proposed positions.

Figure 6 shows āsana for trunk side stretching, trunk twisting and leg outstretching. 

*Parsva Urdhva Hastāsana* (Standing Half Moon Pose or Upward Salute Side Bend Pose) (Figure 6a) and *Bananāsana* (Crescent Moon Pose) variations lying on the side (Figure 6(b1,b2)), *Trikonāsana* (Triangle Pose) (Figure 6(c1–c3)) with extended (*Utthita*) variation (Figure 6(c3)) and *Parivrtta Trikonāsana* (Twisted Triangle Pose) (Figure 6(d1–d3)) are for trunk side outstretch, trunk torsion and leg stretching.

The hip glide, with consequent and wanted hip release, is intense in the proposed āsana.

The involved trunk muscles are the external and internal intercostal muscles, subcostal muscles, transversus abdominis, internal abdominal oblique muscle and internal and external obliques, and a little less the psoas, quadratus lumborum and erector spinae muscles (Figure 6(a,b1,b2,c2)). The posterior muscles of the legs are deeply stretched out (Figure 6(c1–c3,d1–d3)), particularly the posterior thigh muscles (hamstring muscles), namely the biceps femoris long and short head, semitendinosus and semimembranosus. The mainly involved lower leg muscles are the popliteus, gastrocnemius and soleus.

In side bending (Figure 6a,b) and in general always when the center of gravity of a hip is moved laterally (Figure 6a–c), other hip muscles are strongly involved such as the *tensor fascia latae*, *gluteus medius*, *piriformis* (slightly the *gemellus superior* and *inferior* and *obturator internus*) and *internal oblique* abdominals as the hip is moved outside the center of gravity between the feet (in *Parsva Urdhva Hastāsana*, *Bananāsana and Trikonāsana)* (Figure 6a–c) or is pulled toward the posterior foot (*Parivrtta Trikonāsana*) (Figure 6d).

In *Trikonāsana* (Figure 6(c1–c3)), devote attention to *sartorius* muscle overstretching (and lesion); therefore, in case of pain at the internal side of the knee, slightly flex the anterior leg or bend less by moving the support hand from the feet to the tibia. The authors want to attract attention to the different movement, back shape and stretched muscles between the *Trikonāsana* in Figure 6(c2) vs. Figure 6(c3). We differentiate in *Trikonāsana* (Triangle Pose) displayed in Figure 6(c2) in which both trunk sides are rounded with a stretch of intercostal muscles from *Utthita Trikonāsana* (Extended Triangle Pose) illustrated in Figure 6(c3) with straight trunk sides with the following release of the hip corresponding to the back foot.

The execution mode of most āsana depends on the Yoga style. *Trikonāsana* practiced as in Figure 6(c2) follows the majority of Yoga techniques such as Krishnamacharya Yoga (rounded sides trunk and back foot rotated 45 degrees outward) and Yoga from Rishikesh such as *Swami Vishnudevananda Yoga* (1960) and his disciple *Sivananda Yoga* (1969), *Satyananda-Yoga* (1969), etc.

*Trikonāsana* practiced with straight sides trunk and the intention of a deep stretch out of the spine, pushing your head away from your pelvis (two opposing tensile forces; head and pelvis distance each other as much as possible), follow the *Parināma Yoga* technique (and only partially *Iyengar Yoga*).

 

**Figure 7:**
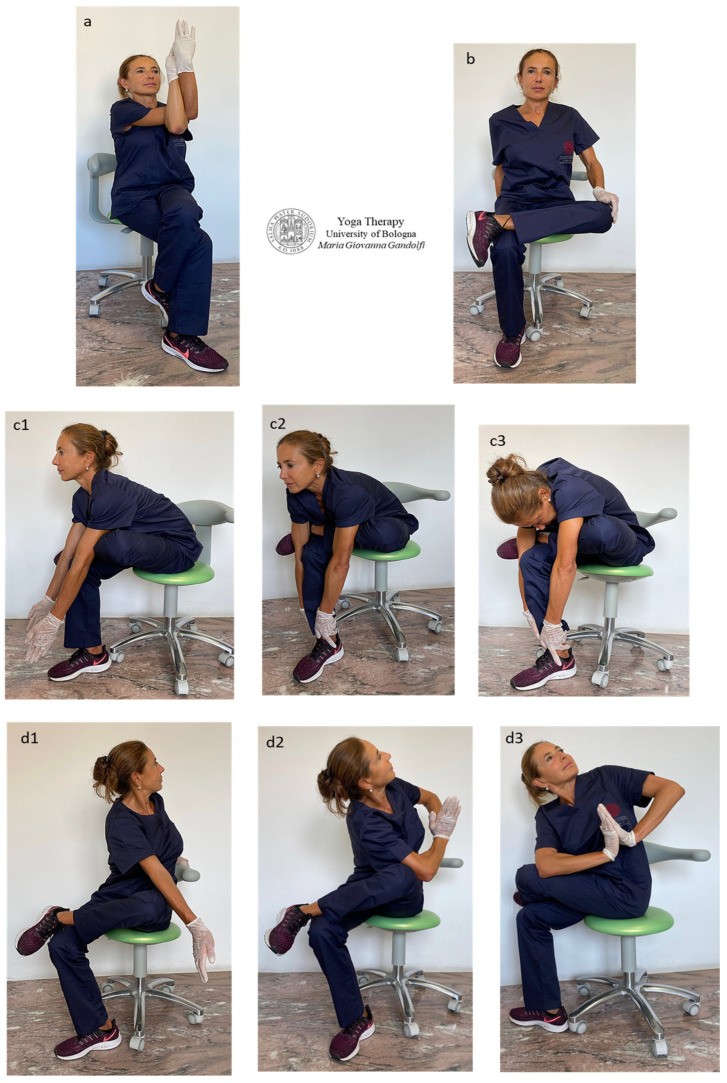
The figure shows selected āsana to release and outstretch hip joint, with following intra-articular decompression. *Garudāsana* seated pose variation (**a**), *Eka Pada Agnistambhāsana* (One-Legged Firelog or Square or Pigeon Pose) seated pose variation (**b**), *Sucirandhrāsana* (Eye of the Needle Pose or Figure-4 Stretch) seated pose variation (**c1**–**c3**), *Eka Pada Parivrtta Utkatāsana* (One-Legged Revolved/Twisted Chair Pose) seated pose variation (**d1**–**d3**). In *Garudāsana* (**a**) and in *Sucirandhrāsana* (**c1**–**c3**), the distraction force and the posterior hip glide at the hip joint can be particularly intense.

Figure 7 displays selected āsana involving movements to release the thigh hip stabilizer muscles (also acting as hip external rotation muscles), namely the *piriformis*, *gemellus superior* and *inferior*, *obturator internus* and *externus*, *quadratus femoris* and *gluteus medius* and *gluteus maximus*.

The visualization of the origin and insertion (specified below in brackets as O ↔ I) of the cited muscles can help to understand the joint compression created by their tightness.

*Garudāsana* seated pose variation (Figure 7a), *Agnistambhāsana* (Double Pigeon Pose or Firelog or Square) seated variation (Figure 7b), *Sucirandhrāsana* (Eye of the Needle Pose, also known as Figure-4 Stretch) seated pose variation (Figure 7(c1–c3)) and *Eka Pada Parivrtta Utkatāsana* (One-Legged Revolved/Twisted Chair Pose) seated pose variation (Figure 7(d1–d3)) are displayed.

Muscles are released and outstretched with the following decompression of the hip joint, particularly the *piriformis* (O: anterior sacrum and sacroiliac joint; I: greater trochanter), *gemellus superior* and *gemellus inferior* (O: ischial spine superior surface; I: greater trochanter medial surface), *quadratus femoris* (O: ischial tuberosity lateral margin; I: intertrochanteric crest), *obturator internus* muscle (O: superior pubic ramus inner surface of the inferior margin and obturator membrane; I: femur greater trochanter), *obturator externus* muscle (O: rami of pubis and ischium, the external bony margin of the obturator foramen; I: femoral neck (trochanteric fossa), *gluteus medius* (O: iliac crest outer surface; I: greater trochanter posterolateral surface) and *gluteus maximus* (O: posterior ilium, posterior sacrum; I: lesser trochanter posterior femoral surface and iliotibial band of *fascia latae*).

The practice of these selected āsana creates a vector force that separates the articular surfaces from each other and represents a joint distraction exercise able to create joint decompression. In *Garudāsana* (Figure 7a) and in *Sucirandhrāsana* (Figure 7(c1–c3)), the distraction force and the posterior hip glide at the hip joint can be particularly intense (the temporary creation of a slight joint gapping at the femoroacetabular joint decreases muscle tightness and outspreads the articulation).

Recommendations for the āsana execution (Figure 7b–d) are to maintain the external rotation of the femur by firmly pressing the ankle onto the knee of the opposite leg (and initially with the help of hand pressure on the knee) (Figure 7b), to roll and anterovert the pelvis to sit on sit bones, to keep lumbar lordosis and to feel equal weight from left to right. Take a deep breath and gradually enter the position, then deepen the movement in sync with the breath. Avoid apnea and fragmented breathing. 

In the āsana with torso torsion (Figure 7(d1–d3)), start the twisting movement with full lungs, starting the twist from the torso and then the head.

## 4. Discussion

This paper describes a Yogāsana protocol specifically ideated for dental professionals consisting of āsana that can be performed in a dental office using a dental stool, a dental unit chair or the dental office walls. The paper would represent a guideline for the self-cure for the prevention or treatment of musculoskeletal disorders affecting dental professionals. 

Our Yoga protocol focused on preventing (or treating) frequent muscle-related syndromes correlated to working activity affecting dental professionals and other workers working in a sitting position such as health professionals like surgeons, sonographers, radiologists and desktop workers. The postures and movements were conceived to mobilize and decompress the musculo-articular system in the low back, hip and legs (including knee and ankle), being areas greatly affected by musculoskeletal disorders. The protocol is based on the joint biomechanics science and on the *theory of bone ties* (joint compressions caused by bone-to-bone tractions through musculo-tendinous-ligamentous system) elaborated on and elucidated in Gandolfi Yoga protocol recently published [9]. The ideated Yogāsana release the bone ties and decompress the joints. It found its rationale in numerous clinical studies demonstrating the effectiveness of selected āsana on low-back and hip disorders.

The main work-related MSDs among dental professionals affecting the lower part of the body include spine disorders (as low-back pain and disc pathologies) and hip compression-related syndromes (*piriformis* syndrome, *quadratus femoris* syndrome, ischiofemoral impingement, femoroacetabular impingement). 

The literature reports that work-related MSDs at the neck and shoulders represent an important problem in occupational medicine. Our recent survey–study reported that 55.1% of dentists and dental hygienists suffer from low-back and hip pain [3]. These disorders add to the cervico-brachial and shoulder pathologies recently described for dental professionals [3,9]: tension neck syndromes and migraine headaches, shoulder disorders, compressive syndromes and neuropathies (carpal tunnel syndrome, De Quervain tenosynovitis, pronator syndrome, thoracic outlet syndrome, impingement syndromes, subacromial impingement).

Protracted static postures and muscle overuse (for working activity or to maintain unbalanced postures) cause the retraction and the tightness of the musculoarticular system/structures. Prolonged sitting, unbalanced and/or twisting working postures, forceful maneuvers and muscle stress are inevitable in daily dental practice. Therefore, the approach to the work-related MSDs must be therapeutic and compensatory.

A base-concept of Yoga Therapy for MSDs is that alterations in muscle length, tone and stiffness cause biomechanical impairments, the reduction in mobility and the increase in compressive forces in the articular system.

Our Yoga protocol indicates precise methods of āsana execution and progression into the movement following the *theory of bone ties* referring to the musculo-tendinous-ligamentous apparatus.

We want to illustrate to the reader the concept that when muscles are tense, they shorten. Shortened muscles add compression to the involved articulation. Shortened muscles force many different other muscles to overwork to perform a movement or to keep the postural balance. Once the muscles become painful, a vicious cycle begins. The pain increases the tension, the soreness, the unbalanced movement and the strain. This worsens the muscle spasm, which in turn increases the pain.

### 4.1. Spine Disorders 

#### 4.1.1. Inclined Forward Posture 

We want to elucidate that the working position of dental professionals obliges to a forward head posture (hyperextension of the upper cervical vertebrae and forward translation of the cervical vertebrae) that means the head is misaligned with the cervical spine, protruding (forward) away from its center of gravity. 

The weight of an adult human head is approximately 5.44 kg (12 lbs.) when aligned on top of the spinal column and the weight increases by 4.54 kg (10 lbs.) for every 2.54 cm (1 inch) displacement forward in the sagittal plane, away from its center of gravity [9]. A small forward displacement of 7–8 cm (3 inches) during dental practice increases the head weight to 18 kg (40 lbs.). The head weight further increases from 18 kg when flexed at 30 degrees up to 27 kg when protruded 60 degrees [53], with a consequent strong increase in muscular efforts in back muscles and isometrically restraining the load of many kilograms against the force of gravity. 

We highlight that the forward head posture of the trunk causes the loss of cervical and lumbar spine lordosis [3].

A recent meta-analysis [54] reports a strong relationship between low-back pain and decreased lumbar lordotic curvature and highlights an increased risk of degenerative conditions/processes of the spine caused by the increased loading and shear forces on the intervertebral disk.

A herniated intervertebral disc of the lumbar spine (i.e., displacement of cartilage, fluid or bone of an intervertebral disc outside the borders of the disc space or its joints) is one of the most serious causes of low-back pain [55]. This condition is frequent in work occupational categories, including heavy load workers [56], sedentary workers [57] and health professionals [58].

A study from a Finnish university group showed a markedly higher L5-S1 lumbar disk degeneration in workers performing heavy occupational physical loading work (30.2%) than low physical loading work (17.1%) (workers, *n* = 1022) [56].

A survey-based study from an Australian university group reported a high percentage of low-back pain (61.2%) in sedentary university employees (*n* = 479) [57].

Health professionals are at a high risk to develop a herniated intervertebral disc of the lumbar spine. A recent survey-based study from a Turkish university group analyzed the occurrence of low-back pain among Otolaryngologists (*n* = 110). The prevalence of low-back pain and disk herniation ranged from 72.4% (doctors involved in surgical procedures, such as rhinoplasty) to 56.5% (doctors not involved in surgical procedures) [58].

A nationwide retrospective study found that younger dentists (*n* = 10,930) (under 34 years) had an almost two times higher risk of cervical herniated intervertebral disc development than a younger general population (*n* = 73,718) [59].

#### 4.1.2. Yoga vs. Disk Herniation and Bulging 

A recent study showed that a Yoga-based intervention reduced neuropathic pain, low-back pain and disability in patients with neuropathic pain due to disc herniation (*n* = 48). The Yoga protocol was 1 h twice weekly for 12 weeks including 16 different listed āsana (unspecified Yoga style) (Easy Pose, Child Pose, Cat–cow, Bridge Pose, Lounge Pose, Pigeon Pose, Tree Pose, Chair Pose, Triangle Pose, Plank, Side plank, Half lord of the fishes, Cow-Faced Pose, Warrior I, Warrior II, Extended side angle) compared with no intervention and only patient education (control) [47].

In a randomized controlled trial from Birmingham University, patients with disc extrusions or bulges (*n* = 61) benefited from Yoga Therapy in both sciatica and nonspecific back pain, with improved pain-related and disability self-reported scores [60]. The Yoga protocol was 30 min at-home daily Yoga exercises (Yoga style not specified) for 3 months compared with no intervention (control). 

A clinical case control study showed that magnetic resonance imaging of long-term Yoga instructors (Yoga style not specified) (*n* = 18) had significantly less lumbar disk degenerative disease than non-Yoga practicing individuals (*n* = 18) [45].

Another study showed improvement in neck pain and disability after therapeutic exercise intervention, consisting in spinal therapeutic exercises (end-range repeated cervical retraction, extension and lateral flexion movements) (10–15 times each session, 6–10 sessions per day for 8 weeks), in patients diagnosed with neck herniated discs, shoulder pain or radiating arm symptoms (*n* = 30) [61]. 

A recent review on the effectiveness of *Iyengar Yoga* in treating neck and back pain (*n* = 6 studies, 570 patients) offered strong evidence for short-term improvements, particularly for chronic spine pain [62].

Yoga is often recommended as an evidence-based additional therapy intervention for back and neck pain. Being more than exercise, Yoga seems to be able to improve body awareness, pain acceptance and coping. One review identified some āsana designated to reduce spinal pain, namely *Tadāsana*, *Ardha Uttanāsana*, Chair *Bharadvajāsana*, *Adho Mukha Virāsana*, *Adho Mukha Svanāsana*, *Utthita Trikonāsana*, *Virabhadāsana II*, *Utthita Parsvakonāsana*, *Prasarita Padottanāsana*, *Supta Padangustāsana*, Prone *Savāsana*, *Supta Pavanamuktāsana*, *Supta Savāsana* [62].

A prospective study from an Indian university showed that selected yogic practices (*Pavanamuktāsana* and Yoga *Nidra*) were effective in reducing pain in participants diagnosed with lumbar spondylitis (*n* = 172). This group was compared to usual physical therapy (control) [63]. The study protocol included 60 min at-home Yoga exercises daily for 4 weeks (*Makarāsana, Bhujangāsana, Sarpāsana, Sarala Salabhāsana, Tadāsana, Tiryaka Tadāsana, Kati Chakrāsana, Ardha Kati Chakrāsana*).

In our Yoga protocol, we propose and describe a technique involving muscles and benefits of many āsana with a backwards bend for back extension, spine mobilization and mainly lordosis recovery and action on the intervertebral disc pressure. Specifically, we described *Upavistha Anuvittāsana* (Seated Backbend Pose) and *Anuvittāsana* (Standing Backbend Pose), *Bhujangāsana* (Cobra Pose), *Urdhva Mukha Svanāsana* (Upward Facing Dog), *Chakrāsana* or *Urdhva Dhanurāsana* (Full-Wheel Pose or Upward Bow Pose) and *Dwi Pada Viparita Dandāsana* (Two-Legged Inverted Staff Pose).

### 4.2. Low-Back Disorders

#### 4.2.1. Low-Back Pain among Dentists

Low-back and hip pain outcomes/results from stiff (intervertebral and hip) articulations, a tight muscle–tendon–ligament–fascia complex and retracted muscles cause the reduction in the articular space and the increase in compressive forces and friction forces, favoring the onset of pathologies such as hip disorders (*piriformis* syndrome, gluteal pain, lateral hip pain, pelvic crossed syndrome or lower crossed syndrome, spine sagittal imbalance, lumbopelvic rhythm alteration, compressive syndromes and neuropathies and spine disorders including lumbar disk herniation, joint inflammation (arthritis) with pain and limited movement or joint degeneration (arthrosis or osteoarthritis) through joint wear and tear over time.

Studies from top-ranked medical journals report that the low back and hip are areas highly affected by musculoskeletal disorders among dental professionals [3,4,5,7,10,11,12]. A high prevalence of low-back pain among dentists has been evidenced in several clinical studies [3,5,7,10], ranging from 48.5% [7] to 64% [10]. 

Several reviews reported a high prevalence of low-back pain in dentists. An Australian research group reports a low-back pain prevalence ranging from 36.3 to 60.1% (21 studies, dentists, *n* = 1428) [11]. A recent systematic review from a Saudi Arabia research group reported a prevalence of 15.7–88.9% (13 studies, *n* = 3443 dentists) [12]. Another recent systematic review and meta-analysis from an Indian research group reported a prevalence of 47.7% (6 studies, *n* = 2098 dentists) [4].

#### 4.2.2. Yoga vs. Low-Back Pain and Spine Disorders

Low-back pain is defined as pain localized between the 12th rib and the inferior gluteal folds, with or without leg pain.

As mentioned in the introduction, the forward bent position of the trunk causes the loss of lumbar lordosis with a strong increase in the intradiscal pressure at the anterior part of the lumbar vertebral discs. The same occurs at the cervical lordosis. This anomalous non-physiological pressure load induces anoxia and activation of metalloproteinases at the anterior portion of the discs and increases the pressure of the nucleus pulposus against the anulus fibrosus that overtime allows disc bulging or protrusion or extrusion with the following discopathies and spine-related compression syndromes.

In our proposed Yoga protocol, we conceived exercises to prevent the loss of physiological spine lordosis or for its recovery and āsana for the release of tight and retracted anterior muscles (hip flexors, anterior thigh muscles, anterior torso muscles) for the prevention of low-back pain, impairment syndromes, *iliopsoas* syndrome and lower crossed syndrome, and to increase low-back lordosis, spinopelvic mobility and lumbopelvic rhythm. In our Yoga protocol, we show and describe different āsana to restore and recuperate the lumbar curve. In particular, *Urdhva Mukha Svanāsana, Anuvittāsana, Bhujangāsana, Chakrāsana, Dhanurāsana and also Salabhāsana* and *Utkatāsana* (Figure 3a–c) increase spine mobility and favor a correct (ergonomic) sitting posture.

It has been demonstrated that Yoga is effective for low-back pain and disc herniation [64,65,66,67,68] when selected Yoga styles have been practiced with a therapeutic aim, such as *Iyengar* Yoga [64,65,66], *Hatha* Yoga [67] or Yoga programs, derived from *Patanjali Yogasutras*, *Upanishads* and *Yoga Vasistha* [68]. 

Several systematic reviews highlighted the beneficial effects of Yoga Therapy regarding the treatment on low-back pain [52,69,70].

Previous systematic reviews on the effectiveness of Yoga to treat low-back pain analyzed a total of 7 and 12 RCTs, respectively. The reviews showed that Yoga exercises have the potential to significantly reduce low-back pain and back pain disabilities in comparison to the control groups provided in the studies (usual care, normal physical exercises or no treatment) [41,52].

Similarly, a more recent systematic review from an American university group analyzed 10 RCTs (*n* = 956 patients) and suggested a beneficial effect of Yoga on nonspecific low-back pain and back-specific disability in midlife adults [70]. The studies included in the review reported different Yoga styles, namely *Iyengar Yoga* (*n* = 3), *Hatha Yoga* (*n* = 2), *Viniyoga* (*n* = 2) and other unspecified ones (*n* = 3) with a treatment duration from 4 weeks to more than 12 weeks [70] 

A multidisciplinary university research group provided guidelines and indicated Yoga as conservative treatments for chronic nonspecific low-back and neck pain [71]. 

A Cochrane systematic review (*n* = 12 randomized clinical trials; 1080 participants) showed that at 3 and 6 months, Yoga improves back-related functions and pain scores [72].

The American college of Physicians also published two articles in Annals of Internal Medicine on Yoga as a conservative nonpharmacologic treatment to treat chronic low-back pain, showing that Yoga moderately improved low-back pain and disabilities when compared to usual therapy [71,73].

A randomized clinical study in Annals of Internal Medicine from the University of Seattle found a higher effectiveness of *Viniyoga* (study duration: 12 months) when compared to conventional stretching and self-care book treatment for chronic low-back pain (*n* = 101 patients) [65]. The Yoga protocol was 45–50 min sessions including breathing exercises, 5 to 11 postures (cobra, knee to chest, wheel, bridge, supine butterfly, extended leg, warrior, standing forward bend, kneeling forward bend, chair) and guided deep relaxation.

Another randomized study published in Annals of Internal Medicine showed that a Yoga program leads to greater improvements in back function for adults with chronic or recurrent low-back pain (*n* = 313 participants) [66]. The Yoga program included 30 min daily 2-times-per-week exercises for 12 weeks (āsana, *pranayama*, relaxation techniques, mental focus and philosophy). The control group included a usual care education booklet for adults with chronic or recurrent low-back pain.

A randomized clinical trial from Karolinska Institute showed that *Kundalini* Yoga and active treatment therapy significantly improved lower sick absenteeism due to low-back pain (*n* = 159 patients) [74]. *Kundalini Yoga* (60 min classes for 6 weeks) included different poses (breath of fire, spine flex, spine flex in Rock Pose, spine twist, bear grip, spine twist with locked elbow, shoulder lifts, neck rolls, alternate bear grip and *Sat Kriya*). An active comparison group received five 60 min strength-training sessions, then at-home exercise twice a week for 6 weeks. A control group received a booklet containing evidence-based advice and no exercises. 

A study from West Virginia University demonstrated functional and pain-related improvements in *Iyengar* Yoga Therapy for patients with chronic low-back pain (*n* = 42 individuals) [64]. *Iyengar* Yoga intervention included a 1.5h class per week (practice of 29 postures using supine, seated, standing, forward bend, twist, and inversion āsana and no back-bending poses) and 30 min at-home Yoga practice 5 days per week for 16 weeks. 

Another randomized study from an Indian university showed that an intensive Yoga program was more effective in reducing pain, anxiety and depression and improving spinal mobility in patients with chronic low-back pain (*n* = 40) when compared to physical exercises and counseling (*n* = 40). The Yoga program (derived from *Patanjali Yogasutras*, *Upanishads* and *Yoga Vasistha*) incorporated various āsana such as *Pavanamuktāsana* and *Bhujangāsana* [68].

A randomized study from an Indian university showed that Yoga Therapy and physical therapy improved back pain and back-related dysfunction of patients with chronic low-back pain (*n* = 70). Yoga Therapy (*n* = 35) included 35 min weekly *Hatha* Yoga classes for 6 weeks, while the control group received conventional therapy (*n* = 35) [67].

#### 4.2.3. *Multifidus* Dysfunctions

The *lumbar multifidus* is involved in stabilizing the lumbar spine, and dysfunction of the *lumbar multifidus* is considered as one important factor leading to low-back pain [75]. 

A prospective study performed in the University of Hong Kong demonstrated that patients with chronic low-back pain (in particular, when a forward stooping posture was adopted) have a reduced cross-sectional area when compared to a healthy control [76]. The cross-sectional changes in the *multifidus* in subjects (*n* = 35) with (*n* = 16) and without chronic low-back pain (*n* = 16) were assessed in four different postures (prone, standing, 25 °C forward stooping and 45 °C forward stooping) [76].

#### 4.2.4. Yoga-Derived Exercises vs. Multifidus Dysfunctions

A randomized clinical trial from a Brazilian university reported an improvement in pain and the disability score that was observed in patients suffering from chronic low-back pain (*n* = 30) performing exercises (30 min, 2 times/week for 6 weeks) focused on stretching of the *erector spinae*, hamstring and triceps surae (*n* = 15) or exercises focused on segmental stabilization of the *transversus abdominis* and *multifidus* muscle (*n* = 15) [77].

In our proposed Yoga protocol, we illustrate āsana with forward bending for back release, namely *Uttanāsana* (Forward Fold Pose), *Pashimottanāsana* (Seated Forward Bend Pose), *Pashimottanāsana* (Revolved Seated Forward Bend Pose), *Adho Mukha Svanāsana* (Downward Facing Dog) and *Upavistha Dwikonāsana* (Seated Double Angle Pose or Yoga Seal Pose) variations.

We also suggest āsana for trunk torsion and trunk side outstretch, such as *Jathara Parivartanāsana* (Belly Twist A or Seated Spinal Twist A) or *Meru Wakrāsana* (Seated Twist), *Parivrtta Utkata Konāsana* (Revolved Goddess Pose), *Parsva Urdhva Hastāsana* (Standing Half Moon Pose or Upward Salute Side Bend Pose), *Bananāsana* (Crescent Moon Pose).

### 4.3. Hip Disorders

#### 4.3.1. Yoga vs. Hip Pain

A recent investigation from the University of Augusta showed that Yoga poses could be effective in improving core endurance and strength and may be a useful treatment strategy for suffering from hip pain (*n* = 30). The study analyzed the trunk and hip muscle activation during different Yoga poses (Chair Pose, High Plank Pose, Dominant Side Warrior Pose 1, Upward Facing Dog Pose) [78].

A study from Rochester University showed that patients with hip pain may experience increased muscular dysfunction during weight-bearing Yoga poses. The protocol included some selected Yoga poses (tree, standing pigeon and warrior II) performed by patients with (*n* = 4) or without hip pain (*n* = 5) [79].

A randomized clinical trial from an Indian University (*n* = 30) showed that in patients with sciatica, *Pawanmuktāsana* and *Ardha Matsyendrāsana* (Yoga style not specified) helped to relieve sciatic pain by toning the pelvic muscles and relaxing the leg muscles, while *Ardha Matsyendrāsana* reduced stiffness and pain in the hip joints and lower extremities [80]. The Yoga intervention was structured as at home, 15–20 min daily for 30 days. 

A randomized controlled trial (RCT) demonstrated that a *Hatha* Yoga program (class 120 min sessions five times per week for 8 weeks) for patients with hip and knee arthritis (*n* = 36) leads to significant improvements in pain relief, muscle stiffness and functions [81]. Different *Yoga* postures were performed, including Mountain Pose, Warrior I and II Poses, Tree Pose, Chair Pose, Easy Seated Pose, Bound Angle Poses, Open Angle Pose, half locust variation, Bridge Pose, standing forward fold, reclining hamstring stretch, reclining twist and Relaxation Pose [81].

A recent randomized clinical trial demonstrated that *Patanjali’s ashtanga* Yoga treatment (120 min, five times per weeks for 8 weeks) showed a significant improvement in health conditions and perceived pain in patients with rheumatoid arthritis (*n* = 66) [82].

Different Yoga postures were performed, including *Trikonāsana, Katichakrāsana, Tadāsana, Virabhadrāsana, Gomukhāsana,***** *Paschim-utaanasana, Shashankāsana, Vakrāsana, Ek-pada-shalabhāsana, Bhujangāsana, Poorna-Shalabhāsana, Makarāsana, Uttanapadāsana, Setubhandhāsana, Pavanmuktāsana, Matsyāsana* and *Savāsana*. 

#### 4.3.2. Hip Osteoarthritis 

Osteoarthritis of the hip (or coxarthrosis) describes a degenerative and irreversible hip condition with cartilage damage with the following increase in friction between the hip socket and the femoral head bones (joint wear). The triggering causes are related to the increase in intra-articular pressure and strain and include frequent excessive (and/or work-related) strain, inflammatory joint disorders, overweight conditions and metabolic disorders.

#### 4.3.3. Yoga vs. Osteoarthritis

A study from one university in the Netherlands showed that patients with hip osteoarthritis (*n* = 203) had lower pain and greater hip functionality and quality of life when submitted to Yoga-derived therapy (30 min, one time per week for 12 weeks of exercises, namely strengthening and improving flexibility exercises around the hip joint, leg and abdominal muscles and aerobic exercises) (*n* = 101 patients) in comparison to usual care (information and counseling) (*n* = 102 patients) [83]. 

A randomized clinical study from an Irish university group demonstrated that patients undergoing total hip replacements had increased pain relief and increased function when performing functional exercises (*n* = 32) compared to usual care (exercise booklet) (control group; *n* = 31). The exercises (Sit-to-stand, Toe raises, Knee raises, Side leg raises, Back leg raises, Single knee bends, Abduction in side lying step ups, Step ups laterally, One-legged standing balance, Advanced one-legged balance, Pelvic raising/lowering) were structured as 35 min, two times per week for 12 to 18 weeks [84].

#### 4.3.4. Hip Impingement

Femoroacetabular impingement syndrome is a hip disorder involving a contact between the acetabulum and the proximal femur inducing degenerative changes and osteoarthritis over time. Femoroacetabular impingement can induce joint stiffness, pain, muscle weakness and impaired performance in active adults.

Ischiofemoral impingement syndrome is a neglected cause of posterior hip pain, which originates from narrowing of the space between the lateral aspect of the ischium and the medial aspect of the lesser trochanter [85]. Femoral anteversion predisposes patients to narrowing of the ischiofemoral space and subsequent *quadratus femoris* muscle injury.

The main symptoms are stiffness, a restricted hip range of motion, hip or groin pain related to movements or positions, pain in thigh, back or buttock areas, clicking and/or catching, locking or giving way and decreased ability to perform activities of daily living and sports. 

Pain can originate (proximally) from the tendinous insertion of the hamstring muscles on the ischial tuberosity distal to its myotendinous junction [85].

This syndrome has a multifactorial etiology and includes prolonged sitting, repetitive hip flexion and rotation movements and repeated stress.

The triggering factors of ischiofemoral impingement are functional causes related to congenital factors or acquired contributors (secondary to hip osteoarthritis or hip fracture) or a positional trigger.

Positional triggers include muscle imbalances of the *abductor/adductor*, *flexor/extensor* and *internal/external rotator* groups:

- uncompensated hip adduction from hip abductor weakness is the greatest functional threat: *hip abductor* weakness allows uncontrolled thigh adduction, which approximates the femur and ischial tuberosity;
- abnormal pelvic tilt; in particular, pelvic retroversion entails flattening of the lumbar spine and sacrum, which move the ischial tuberosities closer to the lesser trochanters;
- *hip abductor* (*gluteus medius*) tendinopathy or damage;
- frequently elicited by prolonged sitting, increased pain with prolonged weight-bearing or sitting or running, exacerbated by long duration of extension, adduction and external rotation of the hip as the distance between the lesser trochanter and the ischium might be reduced.

#### 4.3.5. Yoga vs. Hip Impingement

A study from Harvard University reviewed magnetic resonances of nine patients with hip recurrent pain with the images of healthy patients (*n* = 11), showing that patients with hip pain had a significantly narrower ischiofemoral space and *quadratus femoris*. In addition, the *quadratus femoris* showed edema (100%), a partial tear (33%) and fatty infiltration (8%) [86].

A study from Australian universities analyzed the coordination of deep hip muscles in people with femoroacetabular impingement, showing that individuals with symptomatic femoroacetabular impingement (*n* = 15) had altered coordination of deep hip muscles (*semimembranosus*, *gluteus medius*, *piriformis*, *obturator internus*, *obturator femoris*) during their gait compared to asymptomatic healthy controls (*n* = 14) [87].

A randomized trial from an American university evaluated patients with femoroacetabular impingement (*n* = 80) who underwent arthroscopy surgery (*n* = 66) or eight therapeutical self-mobilization exercises (2–3 sets of 15 repetitions of exercises 1–2 times per day) (*n* = 14). After 2 years, surgical and non-surgical interventions provided a similar outcome in terms of pain, hip activities and perception of improvement [88].

#### 4.3.6. *Piriformis* Syndrome

*Piriformis* muscle connects the femur head with the sacrum and acts as the leg *extra rotator* and *abductor* muscle. *Piriformis* syndrome is caused by compression of the sciatic nerve by the *piriformis* muscle. When there is tight retraction or inflammation, hip pain and stiffness, pain in the gluteal area, posterior pelvic girdle pain and sciatica symptoms are caused. *Piriformis* muscle pain, dysfunction and *piriformis* syndrome are key parts of the differential diagnosis in people suffering from posterior pelvic girdle pain [89].

#### 4.3.7. Yoga vs. *Piriformis* Syndrome

The initial goal of physical therapy is to restore proper length to the muscle and release myofascial trigger points that may be present, hypothetically reducing the compressive force on the sciatic nerve. There are no studies on the effect of Yoga on *piriformis* syndrome, only some recommendations from one study [89] and one study that suggests some physical mobilization exercises. 

A study from a Pakistani university analyzed the efficacy of two distinct stretching techniques (*external rotator* sequences of self-stretching and *abductor* sequence of passive stretching) in females with *piriformis* syndrome (*n* = 30), showing significant improvements in pain reduction and functional performance with both techniques [90]. 

#### 4.3.8. *Quadratus Femoris* Disorders

Limited data are reported regarding the diagnosis and treatment of low-back muscle inflammation or injuries, such as the *quadratus femoris*, *multifidus* and *piriformis*. Alterations of muscle length and tone could alter the lumbar pelvis and may act as additional risks for chronic low-back pain.

A case report on myofascial pain syndrome affecting the quadratus femoris was published by a Spanish research group. Quadratus femoris injury can affect surrounding muscles, being in a close relationship with the ischial tuberosity. A program on stretching and strengthening exercises (not reported in the study) may finalize the proposed surgical approach [91]. 

For all disorders related to the increase in intra-articular pressure and narrowing of the hip joint space (as in hip impingement and hip osteoarthritis, *piriformis* syndrome and *quadratus femoris* syndrome), our proposed Yoga protocol indicates poses for hip distraction and hip glide such as *Trikonāsana* (Triangle Pose), *Garudāsana* (Eagle Pose), *Agnistambhāsana* (Double Pigeon Pose or Firelog or Square), *Parsva Urdhva Hastāsana* (Standing Half Moon Pose or Upward Salute Side Bend Pose), *Bananāsana* (Crescent Moon Pose), *Sucirandhrāsana* (Eye of the Needle Pose or Figure 4 Stretch), *Parivrtta Utkatāsana* (Twisting Chair Pose) and *Eka Pada Parivrtta Utkatāsana* (One-Legged Revolved/Twisted Chair Pose), *Parivrtta Trikonāsana* (Twisted Triangle Pose), and āsana for hip flexors and hip release such as *Eka Pada Supta Virāsana* (One-Legged Reclined Hero Pose), *Parivrtta Virabhadrāsana I* (Revolved Warrior I) or *Parivrtta Ashva Sanchalāsana* (Revolved High Lunge Pose), *Ashva Sanchalāsana* (High Lunge Pose), *Virabhadrāsana I* (Warrior I), *Valakhilyāsana* (Standing Pose of the Heavenly Spirits), *Virabhadrāsana II* (Warrior II), *Viparita Virabhadrāsana* (Reverse Warrior) or *Parighāsana* (Gate Pose).

#### 4.3.9. *Iliopsoas* Syndrome

The process of intervertebral disc degeneration alters the *psoas major* muscle morphology. The *psoas major* originates from the bodies of the lumbar vertebrae, inserts on the lesser trochanter of the femur and is considered as an active postural muscle. 

A clinical study from a Croatian university analyzed through magnetic resonance the changes in the *psoas major* muscle cross-sectional area in healthy patients and in patients with low-back pain. A greater cross-sectional area was shown in low-back pain patients, particularly at the levels of L3/L4 and L4/L5 intervertebral disks [92].

#### 4.3.10. Yoga vs. Psoas Syndrome

There are no studies on the effect of Yoga on the *psoas* disorders.

#### 4.3.11. Hip Imbalances

Pelvic crossed syndrome is a clinical condition characterized by an unbalanced muscle activity, i.e., tightness and overactivity of the hip flexors and low-back extensors and a coexistent underactivity in the *abdominals* and *glutei* create a ‘crossed pattern’ of hindered or altered lumbo-pelvic-femoral movement/balance and loss of sagittal lumbo-pelvic alignment and control [20].

#### 4.3.12. Yoga Exercises for Hip Extension and Gait Function

A clinical study from the University of Virginia [93] showed marked improvements in a hip extension stride length increase and pelvic anteversion reduction in elderly patients (*n* = 23) undergoing a tailored *Hatha* Yoga program. The program was structured as 90 min sessions twice per week and 20 min at-home sessions on alternate days for 8 weeks. Yoga postures included centering, finger and toe weaving, *Virāsana*, *Vajrāsana*, *Tadāsana*, Table Pose, *Salabhāsana*, *Padangusthāsana*, *supta padangusthana*, *uttitha hasta Padangustāsana*, *Supta Baddha Konāsana*, *Eka Pada Bhekāsana* and *Shavāsana*, *Rajakapotāsana*, *Parsvottanāsana*, *Virabhadrāsana* and *Surya Namaskar* [93].

Regarding all disorders correlated with hip flexor retraction (as in *iliopsoas* syndrome, pelvic crossed syndrome and reduced hip extension), our proposed Yoga protocol indicates poses to release hip flexors and to increase low-back extension such as *Eka Pada Supta Virāsana* (One-Legged Reclined Hero Pose), *Virabhadrāsana I* (Warrior I), *Virabhadrāsana II* (Warrior II), *Parivrtta Virabhadrāsana I* (Revolved Warrior I) or *Parivrtta Ashva Sanchalāsana* (Revolved High Lunge Pose), *Ashva Sanchalāsana* (Hihgh Lunge Pose), *Anuvittāsana* (Standing Backbend Pose), *Urdhva Mukha Svanāsana* (Upward Facing Dog), *Bhujangāsana* (Cobra Pose), *Valakhilyāsana* (Standing Pose of the Heavenly Spirits) and *Chakrāsana* or *Urdhva Dhanurāsana* (Full-Wheel Pose or Upward Bow Pose).

### 4.4. Leg Disorders (Knee, Ankle)

#### 4.4.1. Knee Disorders

Patellofemoral pain syndrome is a common source of knee pain due to overloading on the front of the knee.

Contributing factors are tight quadriceps and iliotibial bands, poor hip control and hip muscle disfunction causing excessive patellofemoral joint pressure. There are also muscular imbalances and impairments with tightness of some muscles associated with weakness of other muscles, and it concerns thigh and hip muscles (*quadriceps*, *vastus medialis obliquus*, hip flexors) and also the *gluteus* muscles, *iliotibial band*, *gastrocnemius* and *soleus*. Rehabilitation includes stretches and strengthening exercises and core stability improvement. Recent studies focused on strength deficits of the proximal hip musculature as a contributor to this disorder [84,85,86,87,88,89,90,91,92,93,94,95,96].

#### 4.4.2. Yoga and Yoga-Derived Exercises vs. Patellofemoral Pain Syndrome

A study in a minor journal reports that Yoga Therapy (30 min, six times per week for 4 weeks) significantly reduced pain levels and improved patellofemoral joint-related symptoms and function in patients with patellofemoral pain syndromes (*n* = 63). The Yoga Therapy protocol included *Shithilikarana Vyayama* (loosening exercises), *Tadāsana*, *Dandāsana, Janushirsāsana, Ardha Salabhāsana, Sarpāsana, Utthitāsana, Setubandhāsana, Utkatāsana, Virabhadrāsana I* and *II*, *Makarāsana* [97]. 

A randomized clinical trial from an American university showed a dissipation of pain in patients with patellofemoral pain syndrome (*n* = 33) performing Yoga-derived exercises (seated hamstrings stretch, standing *quadriceps* stretch, *quadriceps* strengthening, standing wall stretch for *triceps surae*) organized in three sessions per week for 4 weeks [94].

In our Yoga protocol, we propose āsana for hip flexor and quadriceps stretching (Figure 4(a1–a4)), for hip flexor release (Figure 4a,c–e and Figure 3b–f), for iliotibial band stretching (Figure 6a–c), for calf and ankle muscles (Figure 2d,e, Figure 3b,c(d1), Figure 4b–f and Figure 6c,d) and for hamstrings (Figure 2a–e and Figure 6c,d). The ideated āsana may prevent or reduce the overloading on the front of the knee and excessive patellofemoral joint pressure due to retracted stiff muscles and muscular imbalances, preventing patellofemoral pain syndrome or ankle tendinitis. In addition, āsana involving ankle mobilization (Figure 2d,e, Figure 3b,c,(d1), Figure 4b–f and Figure 6c,d) provide decompression and outspread of the ankles subjected to repetitive movements of medial rotation and dorsiflexion and plantarflexion of the foot for the use of the dental unit pedal. The devised Yogāsana may prevent ankle joint compression and tendinitis (tibial and peroneal tendons and Achilles tendon).

## 5. Conclusions

A Yoga protocol specifically ideated for dental professionals and focused on the articular decompression and mobilization and muscle release in the lower part of the body is described and shown. Namely, Yogāsana ideated for back detensioning and mobilization, lumbar lordosis restoration, trunk side elongation, hip release and leg stretching and decontraction are shown and described. The project was born from over 10 years of experience of teaching Posturology and Ergonomics to university students of dental disciplines.

The paper would represent a guideline for the self-cure for the prevention or treatment of musculoskeletal disorders affecting dental professionals. Prolonged sitting, unbalanced and/or twisting working postures, forceful maneuvers and muscle stress are inevitable. Therefore, the approach must be therapeutic and compensatory. 

A detailed analysis of work-related disorders affecting the lower body among dental professionals is related, including low-back pain, hip pain and disorders, *piriformis* syndrome and *quadratus femoris* dysfunction (gluteal pain), *iliopsoas* syndrome, *multifidus* disorders, femoroacetabular and ischiofemoral impingement, spinopelvic mobility, lumbopelvic rhythm, impairment syndromes, lower crossed syndrome, leg pain, knee pain and ankle disorders. 

An exhaustive guideline of āsana for MSDs—to decompress low-back and hip joints, release a stiff/steely back and tight hips, soften piriformis and gluteal muscles, recuperate lumbar lordosis and recover spinopelvic mobility and lumbopelvic rhythm—is provided. 

Our devised Yoga protocol indicates precise rules/methods of āsana execution and progression into the movement accordingly with our developed theory of *bone ties* referring to the joint compressions caused by bone-to-bone tractions through the musculo-tendinous-ligamentous system. The ideated āsana soften/decontract the tight muscles and decompress the joints, resulting in recovery of the intra-articular space and joint mobility.

The proposed Yogāsana represent a powerful tool for dental professionals to provide relief to retracted stiff muscles and imbalanced/impaired musculoskeletal structures in the lower body. 

Yoga is a powerful concentrative self-discipline that can act as great support to provide physical well-being in daily life (and business) for dental professionals. 

## Figures and Tables

**Table 1:** Risk factors for sitting behavior. Author elaboration from the Yoga Therapy principles integrated with overall ergonomic recommendations.

✓	Prolonged and unbalanced sitting position
✓	Kyphotic sitting posture
✓	Lumbar lordosis loss in sitting position
✓	Forward trunk inclination
✓	Sacroiliac joint stiffness with alteration of spinopelvic mobility and pelvic tilt (nutation/counternutation)
✓	Tissue restrictions and adaptative responses
✓	Hip muscle restrictions and stiffening
✓	Core muscle support reduction
✓	Diaphragmatic breathing absent or insufficient
✓	Postural kinetics alteration during movements

## Data Availability

Data are contained within the article.
